# Characterizing Multimorbidity Prevalence and Adverse Outcomes in Ethnically and Culturally Diverse Sub-Populations in India: Gaps, Opportunities, and Future Directions

**DOI:** 10.3390/ijerph21030327

**Published:** 2024-03-11

**Authors:** Preeti Pushpalata Zanwar, Robyn Taylor, Tanisha G. Hill-Jarrett, Elena Tsoy, Jason D. Flatt, Zunera Mirza, Carl V. Hill, Arokiasamy Perianayagam

**Affiliations:** 1 Jefferson College of Population Health, Thomas Jefferson University, Philadelphia, PA 19107, USA; 2 Irma Lerma Rangel College of Pharmacy, Texas A&M University, Kingsville, TX 78363, USA; 3 Jie Du Center for Innovation and Excellence for Drug Development, University of Pacific, Stockton, CA 95211, USA; 4 The National Institutes on Aging (NIA) Funded Network on Education, Biosocial Pathways, and Dementia in Diverse Populations (EBDDP), College Park, MD 20742, USA; 5 National Association of Chronic Disease Directors, Decatur, GA 30030, USA; rtaylor@chronicdisease.org (R.T.); zmirza@chronicdisease.org (Z.M.); 6 Memory and Aging Center, University of California, San Francisco, CA 94143, USA; tanisha.hill-jarrett@ucsf.edu (T.G.H.-J.); elena.tsoy@ucsf.edu (E.T.); 7 Global Brain Health Institute, University of California San Francisco & Trinity College Dublin, San Francisco, CA 94158, USA; 8 School of Public Health, University of Nevada, Las Vegas, NV 89119, USA; jason.flatt@unlv.edu; 9 Alzheimer’s Association, Chicago, IL 60601, USA; 10 Social and Economic Survey Research Institute, Qatar University, Doha P.O. Box 2713, Qatar; parokiasamy@qu.edu.qa

**Keywords:** multimorbidity, India, LASI, older adults, chronic disease, non-communicable diseases (NCDs)

## Abstract

India is a large middle-income country and has surpassed China in overall population, comprising 20% of the global population (over 1.43 billion people). India is experiencing a major demographic shift in its aging population. Chronic diseases are common among older adults and can be persistent over the life course, lead to the onset of disability, and be costly. Among older adults in India, the existence of multiple comorbid chronic conditions (i.e., multimorbidity) is rapidly growing and represents a burgeoning public health burden. Prior research identified greater rates of multimorbidity (e.g., overweight/obesity diabetes, hypertension, cardiovascular disease, stroke, and malignancies) in minority populations in the United States (U.S.); however, limited studies have attempted to characterize multimorbidity among older adult sub-populations residing in India. To address this gap, we conducted a narrative review of studies on multimorbidity using the data from the Longitudinal Aging Study of India (LASI), the largest nationally representative longitudinal survey study of adults in India. Our definition of multimorbidity was the presence of more than two conditions in the same person. Our findings, based on 15 reviewed studies, aim to (1) characterize the definition and measurement of multimorbidity and to ascertain its prevalence in ethnically and culturally diverse sub-populations in India; (2) identify adverse outcomes associated with multimorbidity in the Indian adult population; and (3) identify gaps, opportunities, and future directions.

## 1. Introduction

The population of India, with 1.43 billion, recently surpassed that of China. India’s growing population is experiencing a major demographic shift and is rapidly aging [1,2]. With the rapid economic growth in India, and the rapid urbanization of the country, access to Western diets coupled with the rise in aging populations has given rise to multimorbidity, or the presence of two or more chronic diseases. Accelerated aging is occurring in India across age groups [3]. Chronic disease prevention, mitigation, and control in the 21st century is of top importance for high-income and middle-income countries alike. In the U.S., the Centers for Disease Control (CDC) is the public health agency concerned with multimorbidity, while non-communicable diseases (NCDs) are a growing concern to the Ministry of Health and Family Welfare in India (MoHFW) [4,5]. The multimorbidity burden in India is growing due to rapid changes in population demographics related to increases in longevity and because of a greater prevalence of chronic disease risk factors (e.g., lack of physical activity and higher rates of obesity), which are occurring at younger ages [1,2,3]. These same factors (e.g., obesity) have been reported as risks for chronic kidney disease, as well as incident cognitive impairment and its progression to other, more severe stages of neurodegenerative disease, such as Alzheimer’s Disease and Related Dementia (ADRD) [6,7,8].

Prior studies have reported the multimorbidity in India to be as much as 50% or higher among older adults ≥45 years [3,4,5]. Previous studies focused on aging in India have reported that the prevalence of multimorbidity- to increases with age and socioeconomic status (SES) [3,9,10]. For example, the prevalence of multimorbidity as reported in the urban poor sub-population who were ≥75 years old was higher than in the 60–74 age group or the 45–59 age group in India (64.14 vs. 53.41 vs. 33.83) [10].

Many of the chronic and often comorbid conditions (e.g., cancer, cardiovascular disease, stroke) that contribute to multimorbidity generally manifest over the life course and can lead to disability onset, which can pose an economic burden to patients, their families, and the health systems that care for them [6,11,12].

While a prior study has characterized a higher burden of multimorbidity (e.g., higher overweight/obesity, diabetes, hypertension, cardiovascular conditions, stroke, and malignancies) in racial/ethnic minorities among middle-aged and older adults in the U.S. [13], limited studies have characterized multimorbidity among older adults in vulnerable sub-populations residing in India. The rationale for our study was that health-associated adverse outcomes, such as poor quality of life, disability, and mortality, have largely been underexamined among South East Asian Indians residing in India, a middle-income country, in comparison to such examinations among populations in high-income countries. Additionally, in India, public health and medical infrastructure are not as developed—compared to developed countries around the globe, particularly for socioeconomically disadvantaged populations [3,10,14,15]. Multimorbidity can lead to risks of polypharmacy and multiple treatments, which are often provided in a siloed fashion for one chronic condition at a time, as opposed to providing a wholistic care involving a comprehensive geriatric team [3]. Clinical guidelines and medical evidence are also geared towards one disease only and may not be relevant for multimorbid conditions [3]. As a result, multimorbidity often leads to other diseases among older adults, introduced due to side-effects from treatments and due to being cared for by multiple providers [3]. Moreover, health disparity populations who are at risk of multimorbidity are not as well characterized in India as they are in the U.S. The objective of our narrative review was (1) to characterize the definition and measurement of multimorbidity and to ascertain the prevalence of multimorbidity in ethnically and culturally diverse sub-populations within India; (2) to identify adverse outcomes associated with multimorbidity in the Indian adult population residing in India; and (3) to identify gaps, opportunities, and future directions.

## 2. Materials and Methods

The scope of this narrative review and methodology was developed through discussions with a diverse team of funded health disparities, health equity, and minority aging scholars and experts across the U.S. and India.

### 2.1. Health Disparity Populations

We reviewed the (1) 2022 U.S. National Institutes of Minority Health and Health Disparities website [16] and the (2) U.S. Healthy People 2030 website to characterize and identify health disparity populations in the U.S. [16,17]. Health disparity populations were defined as those belonging to various marginalized groups that can be categorized by race/ethnicity, sex/gender, belonging to lower SES status, or underserved rural communities, as well as groups that have historically experienced greater difficulties in accessing health because of their religion, gender, age, mental health status, limitations in cognition, vision, hearing, mobility, geographic location, or any other characteristics that can be associated with systematic discriminatory or exclusionary practices or exclusion [16,17]. We adopted these definitions to identify and characterize health disparity populations in India, recognizing and addressing the limitations of certain constructs within India’s cultural context [16,17]. For example, “race/ethnicity”, a social construct that is relevant in the U.S., does not have a direct equivalent in India. Therefore, in place of race/ethnicity, we utilized “caste”, a group based on social class hierarchy in India, and “religion”, with sub-categories of Hindu, Muslim, Christian, and others, the latter three being minority religions in a secular India [9,18,19].

### 2.2. Rationale for a Narrative Review on Multimorbidity Using the Longitudinal Aging Study of India (LASI) Studies

We utilized published articles using the LASI data for several reasons. First, India is experiencing rapid aging of the population. LASI is a groundbreaking nationally representative longitudinal survey that is designed to collect national data from all of its states and union territories (UTs) from Indian households comprising adults > 45 years, their spouses irrespective of age, family members, and adult children. Data are collected on multiple health domains such as economic, social, and biological indicators. Moreover, each state and UT within India is different in terms of its cultural diversity, literacy, language, and geography. Given the diversity and heterogeneity across states and UTs in terms of languages, culture, food, and geographic location within India, a focus on one country is sufficient, as it provides rich analysis in a unique context. Second, LASI, funded by the U.S. National Institutes on Aging (NIA), was launched with the support of the Indian Government and the Ministry of Health and Family Welfare. LASI is designed based on the U.S. Health and Retirement (HRS) Study [20], allowing for cross-national comparative studies of aging. Third, LASI involves systematic data collection using reliable, consistent and comparable protocols in a national sample of Indians, including villages and rural areas, and has variables on many dimensions of health, social supports, income, and wealth measurements for the rising demographics of older persons and their households in India. The objective of LASI is to provide critical data on India’s growing and culturally unique older population and to examine their health and socioeconomic well-being. These rich, nationally representative data allow for in-depth investigations of the relationship between multimorbidity and other health outcomes, which—can aid in the designing—supportive policies for older adults’ well-being at the regional and national levels in India. Lastly, LASI data are available to researchers from the Gateway to Global Aging website with registration [20].

### 2.3. Search Strategy and Article Selection

Our focused search strategy involved reviewing key emerging papers published in peer-reviewed journals that matched the scope of our objectives. We used the PubMed database to identify key articles using keywords “LASI” AND “multimorbidity” with no date filter, which resulted in 18 articles based on titles, abstracts, and full texts. Studies were included if they were published in English and had any of the following synonymous terms: “multimorbidity”, “non-communicable diseases (NCDs)”, or “chronic disease(s)” in the article title and/or had multimorbidity prevalence on health disparity populations as a topic. We excluded four articles when the articles did not provide the following: (1)—the association of multimorbidity with obesity-related measures, anthropometric indices, and/or physical activity or inactivity; (2)—multimorbidity prevalence in health disparity populations; or (3)—risk or prevalence of adverse health outcomes of multimorbidity. We performed an additional search using “LASI” AND author “Lee J” (principal investigator of LASI) and identified one additional article after reviewing 13 titles and abstracts.

## 3. Results

Our narrative review was based on 15 articles. To address Objective 1, we summarized the findings from eight articles on the definition, measurement, prevalence, and burden of multimorbidity across sub-populations, presented in Table 1 [9,10,14,18,19,21,22,23] and Table 2 [12,18,24,25,26,27,28,29]. Additionally, in Table 2, we summarize the findings of the remaining seven articles that reported the associations between multimorbidity and specific adverse health outcomes to address Objective 2.

Respondents in LASI were asked the following question on multimorbidity: “Has any health professional ever diagnosed you with the following chronic conditions or diseases?”, with binary response options yes and no. Chronic diseases and conditions included the presence of any chronic physical NCDs, chronic mental conditions, and infectious illnesses with a long duration (≥1 year), which may need medical care and can limit activities of daily living (ADLs). Specifically, these included diabetes; hypertension; hypercholesterolemia; thyroid disorder; and diseases of the heart lasting a long period of time, including problems concerning the heart or heart failure or other heart conditions that are prevalent in the long term, such as heart attacks, myocardial infarction and its complications, or stroke. Arthritis or rheumatism, any other bone or joint disease, and conditions including osteoporosis were also part of multimorbidity conditions. Other conditions were diseases and chronic conditions of the lungs, including chronic obstructive pulmonary disease (COPD) or chronic bronchitis, asthma, and cancer or malignant tumor. Neurological conditions that were included in the multimorbidity definition were ADRD; Parkinson’s; or psychiatric diseases such as unipolar or bipolar disorders, depression, and convulsions. Stomach conditions such as gastroesophageal reflux, constipation or indigestion, and stomach cancers were also included. Other diseases that comprised multimorbidity were diseases of the skin or kidneys; urinary incontinence; and oral conditions of mouth, gums, and teeth, including dental caries. Glaucoma/cataracts and hearing impairment were also part of the multimorbidity characterization (Table 1 and Table 2) [1,3,4,5,6,7,9,10,11,12,13,14,15]. In three articles, 16 NCDs coded as binary variable yes and no were included as a measure of multimorbidity to construct a multimorbidity score ranging from 0 to 3, with 0 indicating no multimorbidity, 1 indicating the presence of 1 chronic disease, 2 indicating the presence of 2 chronic conditions or diseases, and 3 indicating the presence of >3 chronic conditions [12,19,25].

Table 1 summarizes the multimorbidity prevalence in India according to age, gender, residence, caste, religion, marital status, living situation, education level, and wealth index within families and across generations. Given the heterogeneity in the operationalization of multimorbidity across studies, the multimorbidity definitions provided in the original articles are reported in Table 1. Across studies, the multimorbidity prevalence increased with age. In those ≥70 years, the multimorbidity prevalence ranged from 25.8 to 47.9. Middle-aged and older women had a higher multimorbidity prevalence compared to men, ranging from 25.5 to 59.6; women living in the northern states of Chandigarh and Punjab reported a higher prevalence (Table 1). Multimorbidity prevalence was more common among the urban residents in India, and these findings were consistent across studies. Among the socioeconomically disadvantaged groups, multimorbidity was common and higher among sub-populations who self-identified as belonging to a lower caste or scheduled caste in comparison to those who reported belonging to scheduled tribes. Additionally, the multimorbidity prevalence was higher in Muslims than in Hindus or among those belonging to other religious groups; higher in those who were divorced, separated, or widowed than in those currently married or never married; and higher in those living alone than those living with a spouse or others. Those with secondary education had a higher multimorbidity prevalence than those with only primary education or no education. The multimorbidity prevalence increased with an increasing wealth index, with the highest prevalence found among those who were the richest, followed by those in the middle, poorer, and poorest wealth indexes (Table 1). Multimorbidity was also prevalent within families. When multimorbidity was found to be present among multiple family members, this type of conjugal multimorbidity was specifically reported between the household head and their spouse. Multimorbidity was also observed between siblings and across three generations (Table 1).

Table 2 also summarizes definitions of morbidity and provides adverse outcomes associated with multimorbidity in older adults residing in India. In LASI, multimorbidity was associated with depression, falls, and oral morbidity; the prevalence of these morbid outcomes was 29, 12.5, and 48.7, respectively, among older adults (Table 2). Multimorbidity was also associated with greater odds of ADRD, poorer late-life cognition, poor ratings on self-reported health, and limitations in the number of key tasks and activities for independence for older persons aging-in-place, as measured by limitations in instrumental activities of daily living (IADL) and activities of daily living (ADL) (Table 2).

## 4. Discussion

Our narrative review of published studies using an NIA-funded LASI cohort synthesized the characterization, definition, and measurement of multimorbidity and the prevalence of multimorbidity in sub-populations of ethnically diverse older adults residing in India (Objectives 1 and 2). We identified health disparity populations who are at risk of multimorbidity and described the prevalence and types of adverse outcomes associated with multimorbidity based on 15 key and emerging papers. While previous research has examined multimorbidity in individual studies utilizing LASI data [9,10,12,14,15,16,17,18,19,21,22,23,24,25,26,27,28,29], our narrative review is a focused synthesis of the current state of multimorbidity in India. Our review highlights specific populations that are vulnerable, with a higher multimorbidity prevalence that can be targeted for intervention and policy to ameliorate the consequences of multimorbidity in India. These health disparity populations can be examined further by using the harmonized global aging data to compare and contrast the prevalence of multimorbidity cross-nationally and in different contexts to better understand the pathways, mechanisms, and consequences of multimorbidity in middle-income vs. high-income countries like the U.S., the United Kingdom, and Mexico. Based on our review, we found that older adults, women, those who report belonging to other backward castes (the most socioeconomically disadvantaged population in India), and Muslims (a religious minority in India) primarily represented health disparity sub-populations in India that are at a disadvantage of a higher burden of conditions that comprise multimorbidity (e.g., diabetes, overweight and obesity, cardiovascular conditions, stroke, and cancer). Additionally, we found that increasing age; geographic factors (e.g., place of residence, residing in urban vs. rural area); economic factors (e.g., education and wealth index); and social factors (e.g., marital status and living situation) all contributed to a higher multimorbidity prevalence. These factors displayed a dose-response gradient; however, the direction of the gradient was different for different factors. For example, those who belonged to richer and the richest categories had higher levels of multimorbidity compared to those who were in the middle-income category or who were poor or poorer. Interestingly, those with higher levels of education had higher multimorbidity than those with lower levels of education. It may be that well-being differences unfavorably influence groups who have methodically experienced more noteworthy impediments to well-being due to their racial/ethnic group; religion; educational attainment; financial status; sexual orientation; age; psychological well-being; mental, tactile, or actual handicap; sexual orientation or gender identity; geographic area; or different qualities generally connected to segregation or prohibition. Previous reports using LASI have suggested that educational attainment may be a surrogate for many other factors such as a higher socioeconomic status, urban living, decreased physical activity, and increased adaptation of a Western diet, which can lead higher HbA1c, a clinical indicator for diabetes and a higher risk of cardiometabolic diseases [11,29].

The disparities in multimorbidity prevalence in Indian sub-populations suggests the presence of several “isms”, such as “ageism”, “casteism”, and “genderism and sexism”, that may operate via similar mechanisms of social disadvantage in India as in the U.S. For example, women are disadvantaged globally in both developed and developing countries -due to lower access to education and subsequently reduced job opportunities and retirement savings [30]. Additionally, women in India may be at a compounded disadvantage due to the persistence of paternalistic cultural values and familial responsibilities to care for family, i.e., children, parents, and in-laws, restricting their entry and growth in the labor market.

Multimorbidity increases the vulnerability and pace of biological aging and the onset of disability. We found that adverse outcomes such as depression and ADRD were common among older adults with multimorbidity, suggesting a high need for interventions that can reduce the risk of these conditions and syndromes among older adults [4]. Prior research has provided well-supported characterizations of mental health as co-occurring with NCDs. The Lancet Psychiatry Commission (2019) recognized this relationship between mental illness and conditions of poor body functioning, highlighting the crucial need for interventions that are focused on physical health to mitigate the multimorbidity burden and achieve the health-related Sustainable Developmental Goals (SDGs) [31]. Depression is a serious public health issue among older adults, as it decreases social participation, cognitive function, and overall quality of life and increases the risk of self-harm, burden of disability-adjusted life years, and overall mortality risk [3,24,31,32]. Community-level, government-supported policy interventions and policies that support the return of intergenerational living are strategies to combat depression among older adults in India [3,24,31,32].

With rising longevity in India, India will experience demographic shifts due to the rapid growth of the older adult population. Because age is a strong factor and determinant for chronic conditions such as ADRD, funding streams focused on health disparity research on ADRD in India are urgently needed [1,2,33,34]. Insights on risk factors for ADRD disparities and on sub-populations that bear the greatest burden of ADRD health disparities are necessary to design and create interventions and implement policy changes for an aging population in India.

### 4.1. Limitations and Strengths

We acknowledge several limitations of our narrative review. First, all original articles that we included were cross-sectional studies using LASI wave 1 data, and definitive conclusions about causal associations of multimorbidity with adverse health outcomes, such as depression, falls, disability, ADRD, and late-life cognition, cannot be made. Second, most studies that we reviewed focused on a single domain (e.g., individual, historical/life course, social, cultural, or structural determinant) of multimorbidity, and the examination of multiple health or healthcare outcomes was limited. We found no articles in our focused review on multimorbidity that addressed environmental factors (e.g., temperature and altitude).

Our study also has multiple strengths. Cross-national comparisons of cardiometabolic risk factors between the U.S. and India have been published by the principal investigators of LASI [11,29]. We included articles examining and reporting investigations of multimorbidity prevalence and adverse outcomes using the NIA-funded LASI cohort. Since systematic data collection and reporting are major capacity-building targets in LMICs, our review was based on LASI, the first comprehensive and nationally representative longitudinal study of older Indian adults incorporating data from all of its diverse states/UTs (except Sikkim). LASI utilized systematic data collection and reporting on several sociodemographic, health, and economic variables in a large aging cohort of Indian adults. We utilized lessons on health disparities from the U.S. and applied them to the Indian population. This contrast provides a unique opportunity to conceptualize multimorbidity among health disparity populations in two widely different middle-income vs. high-income contexts; in India, there are huge differences in education and literacy among older adults, whereas in the U.S., there is higher education and literacy at the population level overall [11,35].

### 4.2. Challenges, Gaps, and Opportunities for Future Directions

We addressed our Objective 3 by identifying several challenges, gaps, and opportunities for future research. First, none of the LASI studies that we examined reported on multimorbidity among those who identify with a third gender category, e.g., those who identify as “hijra”, aravani, or kinnar (i.e., transgender populations), a growing and large segment of the Indian population which is at risk of health disparities due to pronounced discrimination and stigma. Prior research on these populations in India found that these disadvantaged populations experience social rejection and structural stigma, which impacts their physical and psychological health, including through isolation, loneliness, and mental health concerns, thereby increasing broader health disparities [36]. Stigma and the experiences of discrimination, social strain, and accumulated distress may manifest as future diseases. Prior studies have documented that transgender communities in India experience poorer physical and mental health, have significant social strain and distress, and are at a greater risk of social disadvantage (e.g., low income, single, and living alone) [37,38]. Similarly, in the U.S., sexual and gender minority populations experience an increased burden in ADRD risk factors and have been designated as a priority research population by the NIH and the Alzheimer’s Association from a health equity standpoint [39,40]. Second, most included studies in our review reported aggregate findings across India, while state-specific or culture-based sub-group comparisons on multimorbidity were limited. Third, no studies which we reviewed focused on intersectional differences in multimorbidity. For example, the association between lower education and ADRD that was observed in India and the perplexing gradient that was observed between higher education and multimorbidity needs further examination. Additionally, the relationship and pathways between literacy, poverty, rurality, education, and multimorbidity need further examination [11,35].

The NIA Health Disparities framework underscores the interplay of various social and structural factors for the presence of health disparities in the U.S. and the manifestation of these factors as adverse health outcomes in the U.S. [41]. The fundamental source of health inequities in the U.S. can be organized as inequalities in the provision of power and in resources, which comprise the social, economic, and environmental determinants of health [42]. Our group is of the consensus that opportunities for lessons learned from the U.S. and India can inform each country’s efforts to address the multimorbidity burden. For example, identifying, examining, and describing what “isms” are relevant to the U.S. (e.g., racism), what “isms” are unique to India (e.g., casteism) [43], what “isms” are common to the two countries (e.g., genderism/sexism), and what the crossovers or similarities between the two countries are in terms of mechanisms that drive multimorbidity would be worthwhile. For example, are there any gender-related or women-identifying disadvantage(s) due to unequal rights or pay for women in India and/or the U.S.? A better understanding of these questions would require adaptations of well-developed and adapted conceptual frameworks for health disparities from the U.S. to the Indian context, including clear definitions of health disparities and health disparity populations in the Indian context. Adaptations of the conceptual frameworks on social and ecological determinants of health over the life course are needed to identify (1) upstream drivers and root causes of multimorbidity (e.g., unequal allocation of power and resources, which leads to unequal social conditions) and (2) midstream drivers (e.g., systematic environmental factors and historical long-standing policies). These drivers can serve as actionable targets for the community, jurisdiction, and place-based solutions to address downstream health inequities for multimorbidity in health disparity populations in India [34,42,43].

## 5. Conclusions

Multimorbidity is common in older adults in several health disparity populations in India and is associated with multiple adverse outcomes. Given the rapid population aging in India over the next several decades [1], preventing and managing chronic diseases is a 21st century public health priority. To manage multimorbidity, greater and timely access to healthcare is needed. Additionally, affordable and culturally tailored interventions grounded in evidence are required in order to prevent, manage, and control the growing multimorbidity prevalence, especially among disadvantaged groups that lack the means to pay for healthcare. Community partnerships and rapid government policy solutions can be strategically harnessed—for older adult well-being in India and in countries with similar burdens. In summary, our findings and conclusions -only generalize within India, based on the evidence that we have gathered. Future research can examine how the findings from our paper differ for high-income countries, such as the U.S. or countries in Europe.

## Figures and Tables

**Table 1:** Multimorbidity disease definition and prevalence within populations in India.

Study	Population (Setting, Time-Period, Cohort, and Sample Size)	Multimorbidity Disease Definition	Populations with Higher Multimorbidity Prevalence (%)
[18]	LASI, 2017–2018, ages ≥ 45 years and above, n = 65,336	“Cooccurrence of ≥2 chronic health conditions in an individual that persists long-term (i.e., physical NCD, mental health condition or infectious illness). A score was calculated from 12 morbidities that were self-reported by survey respondents”.	“Age group: Overall (27.93), 45–59 (22.63), 60–74 (31.57), **75+ (34.05)**; Gender: **Female (28.93)**, Male (25.57); Residence: Rural (23.27), **Urban (36.43)**; Social Group: **Other Backward Class (28.14)**, Scheduled Castes (23.76), Scheduled Tribes (13.51); Wealth Index: **Richest (38.45)**, Rich (30.87), Middle (26.21), Poorer (24.43), Poorest (18.79); Marital status: **Widowed/Divorced/Separated (30.44)**, Currently Married (26.53), Never married (16.71); Religion: Other (30.03), **Muslim (34.76)**, Hindu (26.17); Education level: No education (24.55), Primary education (30.36), **Secondary education (35.28)**, Higher secondary (32.71); Living Status: **Living alone (29.66)**, Living with spouse and others (26.56), **Living without spouse and others (29.56)**”
[21]	LASI, 2017–2018, across all 35 states (excluding Sikkim) and union territories in India, ≥60 years, n = 31,373	“Chronic morbidity was assessed using the question: Has any health professional ever diagnosed you with the following chronic conditions or diseases? Presence of ≥2 of any of the following conditions: Hypertension, diabetes; cancer or a malignant tumor; chronic lung diseases or chronic lung problems (e.g., asthma, chronic obstructive pulmonary disease/bronchitis); chronic heart diseases or other chronic heart problems (e.g., coronary heart disease, heart attack or myocardial infarction, congestive heart failure); stroke; arthritis or rheumatism, osteoporosis or other bone/joint diseases; any neurological or psychiatric conditions (e.g., depression, Alzheimer’s/dementia, unipolar/bipolar disorders, or convulsions; Parkinson’s); and high cholesterol”.	“Age group: Overall (24.1), 60–69 (22.8), **70+ (25.8)**; Gender: **Female (25.5)**, Male (22.5); Residence: Rural (19.1), **Urban (36)**; Wealth Index: **Richest (35.9)**, Richer (27.9), Middle (22.7), Poorer (20.3), Poorest (16.7); Marital status: **Widowed/Divorced/Separated (30.44)**, Currently Married (23.4), Never married (16); Education level: No education (18.4), Below Primary (26.4), Primary (30.2), **Secondary (35)**, Higher (34.7); Living Status: Living alone (23.5), with spouse (23.3), **with others (25.5)**”
[14]	LASI, 2017–2018, ages ≥ 60, n = 31,464 older adults (rural—20,725 and urban—10,739)	“Chronic morbidity was assessed using the question: Has any health professional ever diagnosed you with the following chronic conditions or diseases? Presence of ≥2 of any of the following conditions: Hypertension, diabetes; cancer or a malignant tumor; chronic lung diseases or chronic lung problems (e.g., asthma, chronic obstructive pulmonary disease/bronchitis); chronic heart diseases or other chronic heart problems (e.g., coronary heart disease, heart attack or myocardial infarction, congestive heart failure); stroke; arthritis or rheumatism, osteoporosis or other bone/joint diseases; any neurological or psychiatric conditions (e.g., depression, Alzheimer’s/dementia, unipolar/bipolar disorders, or convulsions; Parkinson’s); and high cholesterol”.	“The prevalence of multimorbidity was higher in urban vs. rural areas in each sub-category for the following factors: Obesity/Overweight (**48.6** vs. 36.6), High-risk waiste circumference (**49.6** vs. 35.1), High risk waiste to hip ratio (**36.9** vs. 21.0), Physical Activity (**Never**, **36.9** vs. 22.4), Tobacco Consumption (**29.9** vs. 17.1), Alcohol Consumption (**32.9** vs. 15.9), Age group (**70–79 years**, **38.3** vs. 20.4), Gender (**Female**, **37.8** vs. 19.9), Education (**Higher than Secondary**, **38.5** vs. 28.7), Marital Status (**Widowed**, 37.1 vs. 20.3), Working Status (**Not Working**, **42.2** vs. 23.3), Household Wealth (**Richer**, **43.3** vs. 21.2), Caste (**Other Backward Class**, 36.3 vs. 19.9) Religion (**Christian**, **47.9** vs. 25.4)”
[22]	LASI, 2017–2018, ages ≥ 45 years, n = 22,526 families	“Family was referred ≥2 members residing in the same household. Conjugal multimorbidity was defined as presence of multimorbidity among the household head and their spouse; multimorbidity between siblings, or presence of multimorbidity between generations”.	“Family-level multimorbidity (44.46); conjugal multimorbidity (41.8); Siblings multimorbidity (42.86); intergenerational multimorbidity, **three generations (46.07)**. Family-level multimorbidity was largely associated with those residing in urban regions and belonging to affluent class”.
[23]	LASI, 2017–2019, women ages 45–65 years, n = 23,951	“Presence of ≥2 simultaneously. NCD’s included asthma, musculoskeletal disorders, cancer, chronic bronchitis, chronic renal disease, chronic obstructive pulmonary disease, diabetes mellitus, gastrointestinal disorders, chronic heart disease, high cholesterol, hypertension, neurological and psychiatric disorders, obesity, skin disorder, stroke, thyroid disorder, and urinary incontinence”.	“Multimorbidity **(29.8) among women in midlife**. Chandigarh (PR—54.8 per 100 women) and Punjab (PR—52.8 per 100 women) reported the highest prevalence of multimorbidity”.
[19]	LASI, 2017–2018, ages ≥ 45 years and above, n = 59,764	“Self-reported chronic NCDs were collected in response to the question “Has any health professional ever diagnosed you with the following chronic conditions or diseases?” Multimorbidity scores were then generated as one of the simultaneous occurrences of ≥2 chronic diseases. The 16 NCD’s—included asthma, musculoskeletal disorders, cancer, chronic bronchitis, chronic renal failure, chronic obstructive pulmonary disease, diabetes, gastrointestinal disorders, chronic heart disease, high cholesterol, hypertension, urinary incontinence, neurological and psychiatric disorders, skin disease, stroke, and thyroid disease”.	“Age: Overall (28%), 45–49 (17.99), 50–54 (21.82), 55–59 (29.56), 60–64 (30.12), 65–69 (33.67), **70–74 (35.37)**, **75–79 (35.93)**, 80+ (34.22); Gender: **Female (29.62)**, Male (26.07); Residence: Rural (24.30), **Urban (36.55)**; Social Group: Other (34.31), **Other Backward Class (28.51)**, Scheduled Castes (24.03), Scheduled Tribes (14.47); Wealth Index: **Richest (38.22)**, Rich (31.87), Middle (26.80), Poorer (25.27), Poorest (19.76); Current marital status: **Not in Union (30.92)**, In Union (26.97); Religion: Muslim (33.38), Christian (28.75), Hindu (27.06), Others (32.43)”.
[10]	2017–2018 LASI, n = 30,489, urban poor, age ≥ 45 years	“Co-occurrence of—≥2 chronic conditions within an individual, without defining an index disease; 17 self-reported chronic conditions—included hypertension, diabetes, cancer, chronic lung disease, chronic heart disease, stroke, arthritis or bone/joint conditions, psychological or neurological conditions, hypercholesterolemia, thyroid disorder, gastrointestinal problems, skin disease, chronic kidney disease, urine incontinence, oral morbidities, visual impairment, and hearing impairment”.	“Overall: 45.26; Age: **≥75 years (64.14)**, 60–74 years (53.41), 45–59 years (33.83); Gender: **Female (47.31)**, Male (41.06); Caste: **Other backward class (43.30)**, Scheduled Caste (43.07), Scheduled Tribe (38.16); **Monthly Per Capita Expenditure**: **Poorest (47.88)**, Poorer (41.44)”.
[9]	2017–2018 LASI, n = 30,489, age ≥ 45 years	“**Complex multimorbidity** was defined as ≥3 chronic conditions among >2 body systems. These chronic conditions were further catalogued into 11 system- -specific chapters (e.g., neoplasms; endocrine/nutritional/metabolic; mental/behavioral; eye-cataract; glaucoma; ear/mastoid; circulatory system; the digestive system, which includes reflux; oral conditions; respiratory system; skin/subcutaneous tissue; musculoskeletal/connective tissue; and genitourinary system)”.	“Age: Overall (63.25), **60–74 years (47.9)**; Gender: **Female (59.6)**, Male (40.4); Residence: **Rural (59.7)**, Urban (44.3); Social Group: **Other Backward Class (40.4)**, Other Caste (34.0), Scheduled Castes (15.7), Scheduled Tribes (9.9); Wealth Index: **Richest (23.7)**, **Rich (22.1)**, Middle Class (19.9), Poorer (18.6), Poorest (15.7)”.

Bolded refers to sub-populations with high multimorbidity prevalence.

**Table 2:** Multimorbidity prevalence and adverse outcomes of multimorbidity in India.

Study	Study Population and Time Period	Multimorbidity Definition	Multimorbidity Adverse Outcome and Prevalence (%)
[24]	LASI, 2017–2018, age ≥ 60 years, N = 31,464	“An individual diagnosed with ≥2 of the following. 9 different chronic illnesses: hypertension, chronic heart diseases, stroke, any chronic lung disease, diabetes, cancer or malignant tumor, any bone/joint disease, any neurological/psychiatric disease, and high cholesterol. All chronic illnesses were assessed by asking: “Has any health professional ever diagnosed you with the following chronic conditions or diseases?”	“Depression was assessed using the 10-item Centre for Epidemiological Studies Depression Scale (CES-D-10). **Depression among older adults (29)**: Men (26%), Women (31%)”.
[25]	LASI, wave 1, 2017–2019, age ≥ 60 years, N = 28,567	“Determined using 16 self-reported chronic illnesses: hypertension, diabetes, cancer, chronic lung disease, chronic heart disease, stroke, bone or joint diseases, neurological or psychiatric conditions, hypercholesterolemia, thyroid disease, gastrointestinal problems, chronic renal disease, skin diseases, visual impairment, hearing defect, and obesity. Multimorbidity score was constructed as a simple count of all conditions in an individual where each condition was given a score of one”.	“Falls were assessed through the self-reported question “Have you fallen down in the last two years?” reported in binary as ‘Yes’ or ‘No’. **Falls among older adults (12.5)**, Women (13.6), and Men (11.4)”.
[26]	10/66 dementia survey of individuals, 2003–2006, ages ≥ 65 years across urban and rural settings (n = 2004) LASI, 2010, ages ≥ 45 years, (*n* = 386). World Health Organization (WHO) conducted SAGE, a multi-country survey Study of Global Ageing, 2006–2007, ages ≥ 65 years, n = 2441	“Targeted nine potentially modifiable risk factors: less education, **hearing impairment**, **depression**, physical inactivity, **hypertension**, **obesity**, smoking, **diabetes**, and social isolation”.	“Cognitive function was measured using the creation of a cognitive index with a total possible score of 27 and a cognitive z-score based on cognitive tests: verbal fluency in one minute, ten-word learning, and ten-word delayed recall. **Dementia** was calculated by an algorithm developed by 10/66 and Diagnostic and Statistical Manual (DSM) IV research criteria. Less education, **hearing impairment**, **depression**, and physical inactivity were associated with lower z-scores and **increased odds of dementia**”.
[27]	LASI, 2018, age ≥ years, n = 59,764	”Defined based on 12—self-reported chronic physical diseases and conditions (i.e., hypertension, diabetes, cancer, chronic lung disease, chronic heart disease, stroke, arthritis and osteoporosis or other bone/joint diseases, neurological or psychiatric conditions, hypercholesterolemia, thyroid disease, gastrointestinal problems, chronic renal disease) and four other morbid conditions (skin diseases, vision and hearing defect, and obesity). Additionally, multimorbidity was characterized based on physical morbidity present (those who had two or more conditions) or absent, and by body systems”.	“Oral morbidity was self-reported and collected by any one or multiple of the seven specific oral conditions such as, ‘painful teeth’, ‘ulcers lasting more than two weeks’, ‘bleeding gums’, ‘swelling gums’, ‘loose teeth’, ‘dental cavity/dental caries’, ‘soreness or cracks in the corner of the mouth’, and/or any other conditions in the last one year. Other conditions were reported by 51 participants which were ‘not good teeth’, ‘germs problem’, ‘root canal’, ‘heaviness in tongue’, ‘mouth problem’, etc., which were irrelevant and hence were not included in the analysis. Further, oral conditions based on soft tissue and hard tissue conditions were classified. **Oral morbidity (48.56)** and physical multimorbidity (50.36)”.
[29]	LASI–Diagnostic Assessment of Dementia (LASI–DAD) and the Harmonized Cognitive Assessment Protocol of the Health and Retirement Study (HRS-HCAP) in the United States conducted an in-depth assessment of cognition, using protocols designed for international comparison. Respondents aged > 60 years from LASI-DAD (N = 1865) and respondents aged > 65 years from HRS-HCAP (N = 2111) provided venous blood specimen.	“**Cardiovascular System**—Biomarkers included high blood pressure, defined as systolic blood pressure of 140 mmHg or higher or diastolic blood pressure of 90 mmHg or higher; pulse rate; proBNP; and homocysteine. **Metabolic System**—Biomarkers included BMI based on measured height and weight (self-reported if measured height and weight are not available), HbA1c, HDL cholesterol, and lipoprotein”.	“For cross-country comparisons, a harmonized total cognitive score and a summary score based on the following tests were used to provide a comprehensive assessment of various cognitive domains: 10-word learning, including immediate and delayed recall and recognition 12; logical memory (i.e., Brave Man story only), including immediate and delayed recall and recognition 13; Mini-Mental State Examination (MMSE) 14 or a validated Hindi version of MMSE summary score 15; verbal fluency score, 16 which was the number of named animals within 60 s; the community screening instrument for dementia score 17; and the Raven test, 18 a count of the number of correct answers to a series of images that required the respondent to select the missing piece. The range of total cognitive scores was 0 to 175. In both U.S. and India, lower **late-life cognition** was associated with older age, lower education, **elevated homocysteine**, **elevated proBNP**, and **lower albumin levels**. In India, the associations between HbA1c levels and cognitive measures were positive, with a coefficient of 1.5 (*p* < 0.001)”.
[28]	LASI, 2017–2018, n = 65,562, age ≥ 45 years	“Measured as the presence of ≥2 morbid conditions, including hypertension, chronic heart diseases, any chronic lung disease, diabetes, any bone/joint disease, and cancer”.	“Self-rated health (SRH) was assessed based on the question “Overall, how is your health in general? Would you say it is very good, good, fair, poor, or very poor?” The dichotomized version of the poor SRH variable was constructed such that “poor” and “very poor” were coded as “Yes;” whereas “very good,” “good,” and “fair” were coded as “No”—The odds of reporting **poor SRH among older adults with more than one morbidity were 3.78 times higher** [AOR: 3.78, 95% CI: (3.54,4.04)] than adults with no morbidity”.
[12]	LASI, 2017–2018, age ≥ 60 years, n = 31,464	“The number of chronic conditions and diseases were self-reported and assessed through the question “Has any health professional ever diagnosed you with the following chronic conditions or diseases?” Chronic conditions included hypertension, diabetes, neurological/psychiatric disease, lung disease, heart disease, stroke, and bone-related disease. Further, the number of chronic diseases was categorized into no disease, single, two, and three plus based on the number of reported diseases”.	“Activities for Daily Living (ADL) and Instrumental Activities of Daily Living (IADL) were coded high and low, with high representing no difficulty in ADL/IADL and low representing a difficulty in at least one ADL/IADL. 1. ADL referred to typical daily self-care activities (such as movement in bed, changing position from sitting to standing, feeding, bathing, dressing, grooming, personal hygiene); ability or inability to perform ADLs was used to measure a person’s functional status in people with disabilities and older adults. 2. IADL functions were those that let an individual live independently and live and function in the community. Respondents were asked if they had any difficulties that were expected to last more than 3 months, such as with preparing a hot meal, shopping for groceries, making a telephone call, taking medications, doing work around the house or garden, managing money (such as paying bills and keeping track of expenses), and getting around or finding an address in unfamiliar places. Low ADL and IADL were 2.16 (CI: 1.71, 2.72) and 2.89- (CI: 2.067, 4.05-) times higher among older adults with >3 chronic conditions”.

Bolded refers to adverse outcomes of multimorbidity.
